# A Study on High-Rate Performance of Graphite Nanostructures Produced by Ball Milling as Anode for Lithium-Ion Batteries

**DOI:** 10.3390/mi14010191

**Published:** 2023-01-12

**Authors:** Vahide Ghanooni Ahmadabadi, Md Mokhlesur Rahman, Ying Chen

**Affiliations:** Institute for Frontier Materials, Deakin University, Waurn Ponds, VIC 3216, Australia

**Keywords:** lithium-ion batteries, anode, graphite, specific surface area, rate capability

## Abstract

Graphite, with appealing features such as good stability, high electrical conductivity, and natural abundance, is still the main commercial anode material for lithium-ion batteries. The charge-discharge rate capability of graphite anodes is not significant for the development of mobile devices and electric vehicles. Therefore, the feasibility investigation of the rate capability enhancement of graphite by manipulating the structure is worthwhile and of interest. In this study, an effective ball-milling process has been set up by which graphite nanostructures with a high surface area are produced. An in-depth investigation into the effect of ball milling on graphite structure as well as electrochemical performance, particularly rate capability, is conducted. Here, we report that graphite nanoflakes with 350 m^2^ g^−1^ surface area deliver retained capacity of ~75 mAh g^−1^ at 10 C (1 C = 372 mA g^−1^). Finally, the Li^+^ surface-storage mechanism is recognised by associating the structural characteristics with electrochemical properties.

## 1. Introduction

Electrode materials with high energy and power densities, as well as low cost and scalable preparation methods, can open up opportunities for the development of high-performance lithium-ion batteries (LIBs) for powering mobile devices and low-emission vehicles such as hybrid electric vehicles (HEVs), plug-in HEVs, and electric vehicles (EVs) [1]. Another significant demand for future LIBs is the fast-charging capability of the electrode materials, especially anode materials for EVs, to reduce charging time compared with the fuelling time of the existing vehicles [2,3,4].

In 1991, using graphite anodes with a flat potential profile below 0.5 V vs. Li/Li^+^ and a considerable specific capacity of 372 mAh g^−1^ (corresponding to one lithium per hexagonal carbon ring, i.e., LiC_6_) led to increasing the volumetric and gravimetric energy density of LIBs up to 400 Wh L^−1^ and 165 Wh kg^−1^, respectively [5]. Additionally, graphite offers other advantages of good stability, high electrical conductivity, non-toxicity, low cost, low volume change during lithiation (only 10%), and natural abundance. Therefore, graphite was used as a good alternative to soft/hard carbons, and it is still the most common anode material in LIB technology [6]. Basically, graphite is composed of graphene layers held together by van der Waals forces in a stacking sequence of AB or ABC, while Li-ions intercalation into these layers results in an AA stacking configuration [7]. The other structural feature of graphite is that carbon atoms make an sp^2^-hybridisation leading to a delocalised electron network in hexagonal graphene layers, thus creating high electrical conductivity [8]. Despite the valuable features of graphite anode in LIBs, the high-rate performance of graphite is limited, and this issue has not been addressed as much as capacity.

Many research efforts have already been devoted to enhancing the performance of graphite in LIBs. One strategy is the creation of new graphitic structures such as carbon nanotubes [9] and graphene [10,11,12]. The other approaches are manipulating graphite structure through doping non-metal elements (B, N, P, S) [13,14,15,16] or disordering graphite structure by various ball-milling techniques [17,18,19,20,21]. It is demonstrated that the rate capability of the electrode materials in LIBs depends critically on the migration speed of Li^+^ ions and electrons through the electrolyte and bulk electrodes. Employing nanomaterials with reduced path length can accelerate electrode kinetics [22,23]. Among various techniques, ball milling is recognised as a suitable method, which can produce a large number of materials and effectively reduce the particle size down to the nanometre level.

In 2001, Natarajan et al. [21] examined the effect of ball milling on the lithium intercalation properties of graphite powder and found that the reversible capacity of the milled graphite powder decreases, but irreversible capacity increases with increasing milling time. This behaviour was attributed to the destroyed surface and increased basal plane surface area due to extended milling, which leads to the exfoliation of the graphite by intercalation of the solvated lithium ions. However, a comprehensive electrochemical investigation is required to study the structure-LIB function relationship of the ball-milled graphite.

Later on, Robledo et al. [24] performed ball milling of graphite for long durations under an oxygen atmosphere, which resulted in an increase in the Li^+^ storage and capacity of the graphite electrodes. They correlated this behaviour to the oxygen content of the milled graphite, which increases with milling time and provides oxygen-containing species as the adsorption sites for Li^+^ ions. However, the demonstrated capacity (250 mAh g^−1^ at 0.5 C) and rate capability (~50 mAh g^−1^ at 10 C) of the milled graphite was not very promising.

Recently, Xing et al. [25] reported that the capacity of ball-milled graphite in an argon atmosphere increases with increasing milling time due to the disordering of the graphitic structure, creating a porous structure. Even though they presented higher capacity and rate capability for milled graphite compared to the commercial graphite, their obtained capacity for commercial graphite at a low current density of 37 mA g^−1^ was ~225 mAh g^−1^, which is far lower than the theoretical capacity of graphite. Consequently, the capacity (~316 mAh g^−1^ at 0.1 C) and rate capability (~20 mAh g^−1^ at 5 C) of the ball-milled graphite were rather low. In their study, however, a new capacitive reaction mechanism (rather than the typical lithium intercalation mechanism) was proposed.

In this study, a facile but effective ball-milling process is designed by which graphite nanostructures are produced from commercial graphite, and a positive linear correlation between ball-milling duration and surface area of graphite is obtained. The structure and morphology of the produced nanostructured graphite powders with a high surface area are studied using XRD, BET, Raman, SEM, and TEM analysis. An in-depth investigation of the electrochemical behaviour, especially the rate capability of nano-graphite powders, is conducted to achieve a good overview of the LIB performance of the graphite nanostructures. Finally, the mechanism behind the LIB performance of the nanostructured graphite electrodes at low and high current rates is proposed.

## 2. Materials and Methods

### 2.1. Materials Preparation

Commercial graphite (CG) powder (particle size <20μm, 99% purity, Sigma-Aldrich, NSW, AU) was used as the starting material for producing ball-milled graphite using a Fritsch PULVERISETTE7 premium line machine. Then, 2.5 g of CG powder was loaded into the ball-milling jar, and the milling process was carried out in an Ar atmosphere using 1.25–1.6 mm zirconia microbeads with a ball-to-powder ratio of 28:1 and a rotation speed of 600 rpm. After milling, the extraction of materials from the milling jar was performed in a nitrogen glovebox. A series of materials was prepared, and samples were denoted by their milling duration of 1, 2, 4, and 5 h.

### 2.2. Structural Characterisations

X-ray diffraction (XRD) experiments were carried out using a PANalytical X’pert pro instrument with Cu Kα X-ray of λ = 1.54181 Å, an operating voltage of 40 kV, and a 30 mA current. Brunauer–Emmett–Teller (BET) specific surface area was obtained by a 5-point method and N2 adsorption at 77 K measured by Tristar 3000, Micromeritics Ltd. (Norcross, GA, USA) instrument. Raman spectroscopy characterisation was performed by a Renishaw inVia micro-spectroscopic system with a laser wavelength of 514 nm. Scanning electron microscopy (SEM) images of the powders were acquired from a Hitachi S4500 Zeiss Supra 55VP instrument operated at 2 kV. Transmission electron microscopy (TEM) was employed for obtaining TEM bright-field images using a JEOL JEM 2100 instrument operating at 200 kV with the LaB_6_ source beam, a Gatan Orius camera, and a Gatan Digital Micrograph software V3 (Gatan Inc., Pleasanton, CA, USA). Microstructural Image Processing (MIP) software was used for the quantitative analysis of SEM and TEM images of graphite powders.

### 2.3. Electrochemical Measurements

In order to prepare test electrodes for electrochemical measurements, commercial graphite or ball-milled graphite, carbon black, and PVdF (polyvinylidene fluoride) in a weight ratio of 8:1:1 were mixed thoroughly and dissolved in NMP (*N*-methyl-2-pyrrolidone). The slurry was uniformly coated onto the copper foil of 1 × 1 cm^2^, and the electrodes were dried in a vacuum oven at 90 °C overnight. CR2032-type coin cells containing the prepared test electrode, Li metal as a counter electrode, and a microporous polyethylene film (MTI Corporation, Richmond, CA, USA) as a separator were assembled in an argon (Ar) glovebox. The electrolyte was 1 M LiPF_6_ salt dissolved in a mixture of ethylene carbonate (EC), dimethyl carbonate (DMC), and diethyl carbonate (DEC) by a 1:1:1 volume ratio.

The galvanostatic charge-discharge capacities were measured for the coin cells using a Land battery testing CT2001A system (Wuhan Land Electronics Co. Ltd., Wuhan, China), and the data were collected by LANDdt software V7 (Landt Instruments, Vestal, NY, USA). The charge-discharge tests were conducted under different current densities within the voltage range of 0.01–2 V. Cyclic voltammetry (CV) measurements were performed on the cells in the voltage range of 0.01–2.0 V at a scan rate of 0.05 mV S^−1^ via a Solartron Analytical Electrochemical Workstation (1470E cell test system, Hampshire, UK).

## 3. Results and Discussion

### 3.1. Structural Characterisation

**Figure 1:**
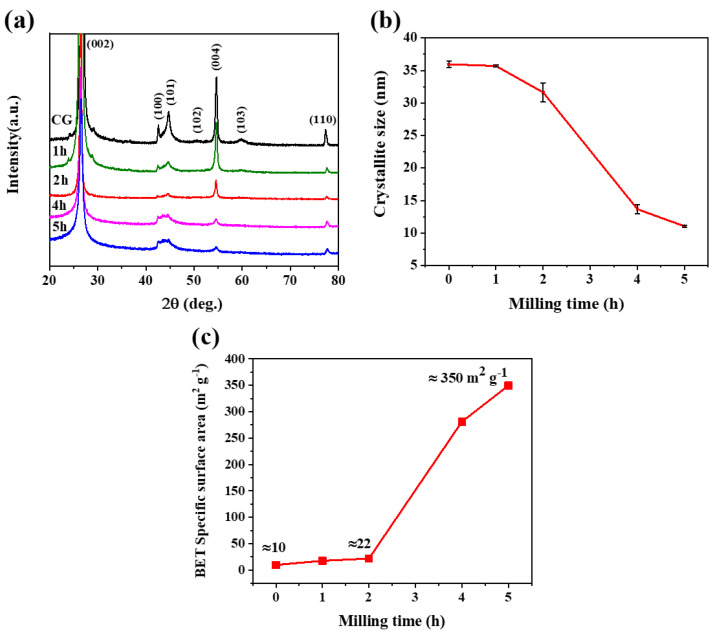
XRD patterns (**a**); crystallite sizes (**b**); BET-specific surface area (**c**) of commercial graphite (CG) and ball-milled graphite samples.

XRD patterns of the commercial graphite (CG) and ball-milled graphite are shown in Figure 1a. All the pronounced diffraction peaks for the ball-milled samples are well-matched with the commercial graphite peaks. The average crystallite sizes of the CG and ball-milled samples were calculated using the Scherrer formula:

(1) $$L=\frac{K\lambda }{\beta .\mathrm{Cos}\theta }$$

where *K* is a constant related to crystallite shape normally taken as 0.9, *λ* is the X-ray wavelength in nanometres, and *β* is the peak width of the diffraction peak profile at half maximum height in radians [26]. Figure 1b shows that crystallite size decreases with increasing milling time, as it is the normal phenomenon in the ball milling of crystalline materials.

The specific surface areas of the commercial graphite and ball-milled graphite were analysed using the Brunauer–Emmett–Teller (BET) method. Commercial graphite (CG) exhibits a much smaller BET-specific surface area of 9.75 m^2^ g^−1^ as compared to those of the ball-milled 2 h (21.73 m^2^ g^−1^), 4 h (281.21 m^2^ g^−1^), and 5 h (349.57 m^2^ g^−1^) samples (Figure 1c). BET surface area shows no considerable increase when ball milling is performed in a short time (1 h). It is, however, clearly seen that BET surface area increases with increasing milling time; therefore, the applied ball-milling conditions are appropriate to prevent welding particles together after milling for certain hours. The sample of 5 h ball-milled exhibits the highest surface area of approximately 350 m^2^ g^−1^, which is about 20 times higher than that of the 1 h ball-milled sample at around 18 m^2^ g^−1^.

**Figure 2:**
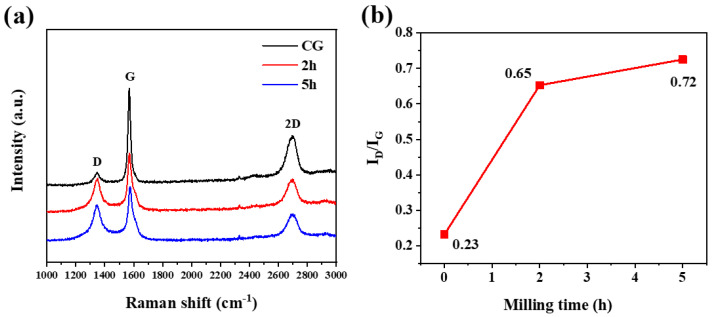
Raman spectra (**a**) and ID/IG (**b**) of CG, 2 h, and 5 h.

Figure 2a shows Raman spectra of CG and milled graphite. The spectrum of CG has 3 regular bands of D, G, and 2D at positions of around 1346, 1569, and 2695 cm^−1^, respectively. The intensity of the D band is lower for the CG, demonstrating a relatively good graphitic structure [27]. By milling commercial graphite for 2 h, D band intensity rises significantly; however, by further milling to 5 h, no major alteration is found in D band intensity. The intensity of the G band related to SP^2^ carbon networks [28] decreases with ball milling. This indicates the reduction of SP^2^ domains in the basal planes. On the other hand, the 2D band is very sensitive to the stacking order of the graphene sheets along the c axis [29]. Ball milling decreases 2D band intensity, which corresponds to the change in the original order of the basal planes and creates a stacking fault in the graphite structure. The intensity ratio of I_D_/I_G_, which is an indication of structural defects quantity [30], rises with increasing milling time (Figure 2b). The intensity ratio of 0.23 in CG changes rapidly to 0.65 after milling for 2 h, and it is then increased slowly. The variation of I_D_/I_G_ demonstrates the creation of structural defects in the ball-milled graphite; however, the growth of structural defects by further milling from 2 h to 5 h is not as major as that of 2 h milled graphite.

**Figure 3:**
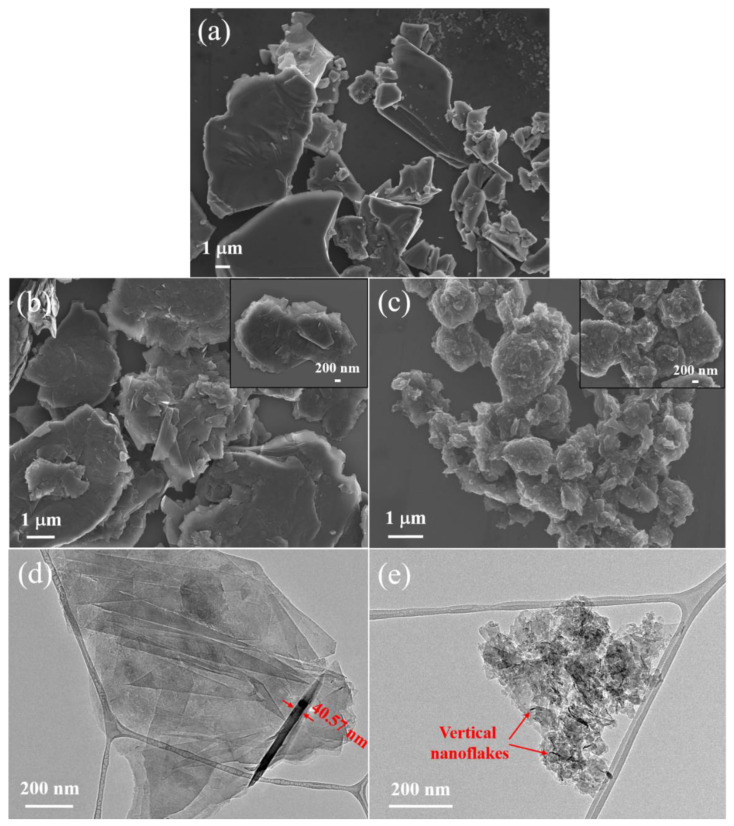
Electron microscopy characterisation of CG and ball-milled graphite: low and high-magnification SEM images of CG (**a**), 2 h (**b**), and 5 h (**c**); TEM images of 2 h (**d**) and 5 h (**e**).

Figure 3 shows the SEM and TEM images of CG and ball-milled graphite. As can be seen in Figure 3a, numerous large sheets of CG with the size of 0.5–17 µm form CG clusters. A significant morphological alteration is visualised in the milled samples. Milling CG for 2 h leads to exfoliating the graphite layers to nanosheets with a length of 140–800 nm, which are further agglomerated and form several micrometre-clusters (Figure 3b,d). TEM image of 2 h milled graphite clearly represents the polygonal morphology of the graphite nanosheets with a thickness of 34–53 nm (through thickness measurement of the perpendicular nanosheets to the page). By increasing the milling time to 5 h, graphite flakes change into agglomerated fine graphite particles with a size of 20–180 nm (Figure 3c,e). The TEM image reveals graphite flakes appearing in the form of nanoflakes with a thickness of 7–20 nm by ball milling for 5 h. High-magnification TEM images of the 5 h sample are represented in Figure S1.

### 3.2. Electrochemical Characterisation

**Figure 4:**
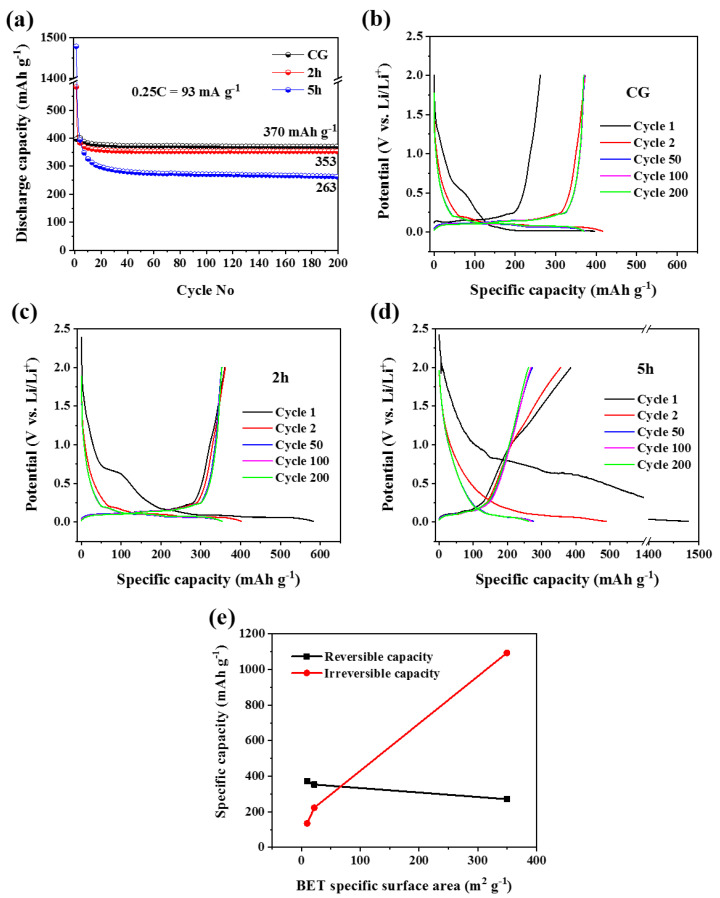
Electrochemical performances of CG, 2 h and 5 h electrodes: (**a**) cycling stability at a 0.25 C (1 C = 372 mA g^−1^) up to 200 cycles; (**b**–**d**) corresponding galvanostatic discharge/charge profiles for the selected cycles obtained at 0.25 C; (**e**) reversible and irreversible capacity as a function of BET specific surface area of the active material.

Figure 4a compares the cycling performance among the three electrodes of CG, 2 h, and 5 h at a current density of 0.25 C (1 C = 372 mA g^−1^) within the voltage range of 0.01–2.0 V. The obtained discharge capacity of the CG electrode is ~370 mAh g^−1^ after 200 cycles, which delivers capacity retention of 99.46% with respect to the theoretical capacity of 372 mAh g^−1^. Ball-milled 2 h electrode exhibits a stable capacity of ~353 mAh g^−1^ (~95% retention with respect to the theoretical capacity of 372 mAh g^−1^) after 200 cycles, whereas it is ~263 mAh g^−1^ for the 5 h electrode. Ball-milled 5 h electrodes exhibit lower capacity retention than that of 2 h and CG electrodes. Cycling performance of the ball-milled 1 h and 4 h electrodes is depicted in Supplementary Materials (Figure S2a) with a lower capacity retention than CG. It is demonstrated that capacity retention of the milled graphite electrodes decreases with increased milling time at a current density of 0.25 C.

The corresponding discharge/charge voltage profiles of CG, 2 h, and 5 h electrodes are shown in Figure 4b–d. The measured 1st, 2nd, 50th, 100th, and 200th cycle discharge/charge capacities were found to be around 396/262, 416/373, 373/371, 371/371, and 370/370 mAh g^−1^ for CG electrode; 583/360, 402/362, 353/352, 353/352, and 353/352 mAh g^−1^ for 2 h electrode; 1479/385, 489/356, 277/273, 271/269, and 263/262 mAh g^−1^ for 5 h electrode, respectively. Considering the charge curves obtained at the end of each cycle and capacity retention at the charge, the 2 h milled graphite electrode demonstrates the best reversibility.

In discharge/charge curves of the CG, 2 h and 5 h electrodes, CG electrode shows typical flat discharge/charge curves with the majority of capacity between 0.1–0.25 V vs. Li/Li^+^ (Figure 4b), whereas 5 h electrode shows sloppy curves with small plateaus, leading to capacity spreading over 0.01–2.0 V (Figure 4d). In the case of the 2 h electrode, discharge/charge curves still show a significant plateau within the same voltage range, similar to the CG electrode with a shorter capacity range but better reversibility than the CG electrode (Figure 4c).

The BET surface area is increased dramatically when milling time is increased from 2 h to 5 h, leading to the high irreversible capacity of the electrodes but decreasing reversible capacity (Figure 4e). The same trend is also observed in 1 h and 4 h electrodes, as shown in Figure S2b.

**Figure 5:**
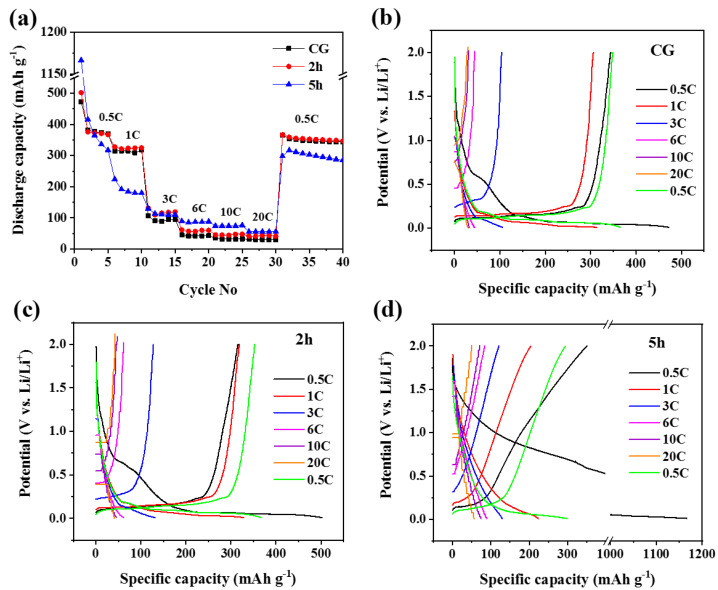
Rate performance (**a**) and corresponding discharge/charge potential profiles obtained at each rate (**b**–**d**) of the CG, 2 h, and 5 h electrodes.

Multi-current galvanostatic discharge/charge experiments were carried out to achieve a deep understanding of the effect of milling time on the rate performance. The consecutive cycling behaviour at different discharge/charge rates was measured after 5 cycles in ascending steps from 0.5–20 C (1 C = 372 mA g^−1^) and followed by a return to 0.5 C (Figure 5a). It is seen that both 2 h and 5 h electrodes show higher reversible capacity at high current rates of 3–20 C than that of the CG electrode. Even though CG and 2 h electrodes exhibit similar electrochemical performance in the moderate current rates of 0.5 and 1 C, the rate performance of 2 h electrodes at a high current rate of 3–20 C is higher. The reversible capacity of the 5 h electrode in moderate current rates (0.5 and 1 C) is much lower than that of 2 h and CG electrodes, and the results are well consistent with the cycling performance, as shown in Figure 5a. However, an interesting electrochemical phenomenon of the 5 h electrode is observed when the current rate increases from moderate to very high. The 5 h electrode delivers a reversible capacity of 86.8 mAh g^−1^ at 6 C, 74.3 mAh g^−1^ at 10 C, and 55.7 mAh g^−1^ at 20 C, which is higher than that of CG and 2 h electrodes showing 42.1 and 60.2 mAh g^−1^ at 6 C, respectively. Such a high-rate performance of the 5 h electrode could be related to the new lithiation/de-lithiation mechanism. It is visualised in the corresponding discharge/charge potential profiles as no longer typical plateau is observed in the curves of the 5 h electrode at high current rates of 3–20 C (compared to 2 h and CG electrodes) (Figure 5b–d).

**Figure 6:**
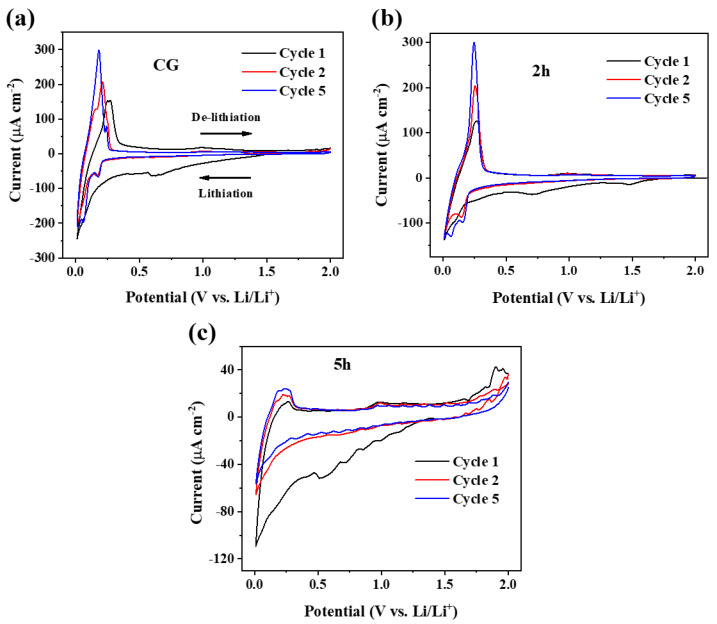
Cyclic voltammograms of CG (**a**), 2 h (**b**), and 5 h (**c**) electrodes with a scan rate of 0.05 mA s^−1^.

To further assess the electrochemical behaviour of Li^+^ ions with CG, 2 h, and 5 h electrodes, cyclic voltammetry (CV) studies were carried out at a scan rate of 0.05 mA s^−1^ in the voltage range of 0.01–2.0 V (Figure 6). For the CG electrode, a pair of typical redox peaks are observed in the CV curve (Figure 6a). In the cathodic scan, the voltage of lithium insertion (intercalation) is about 0.18 V vs. the Li/Li^+^ reference electrode, whereas, in the anodic scan, the voltage for lithium extraction (de-intercalation) varies between 0.18–0.27 V in different cycles [31]. In addition, a broad peak is seen at around 0.62 V in the first cycle cathodic scan, which corresponds to the SEI (solid-electrolyte interphase) layer formation. For the ball-milled graphite electrodes, the CV profiles change significantly. The CV curve of the 2 h electrode demonstrates that lithium can smoothly intercalate and de-intercalate into the electrode (Figure 6b). A pair of dominant redox peaks at around 0.06, 0.14 V/0.25 V is clearly observed in the cathodic/anodic scan, which corresponds to the intercalation/de-intercalation process. The reversibility is quite good for the 2 h electrode as it shows very strong and overlapping de-intercalation peaks. Two peaks at around 0.75 and 1.5 V vs. Li/Li^+^ are observed in the first cycle in the 2 h electrode. The peak at 0.75 V is related to the SEI layer formation; however, the additional small peak at 1.5 V is unknown for this electrode. It is apparent that the ball-milled 5 h electrode performs differently in the CV experiment (Figure 6c). In the CV of the 5 h electrode, the intensity of the typical intercalation/de-intercalation peaks is low, which implies that the intercalation/de-intercalation mechanism is no longer the dominant mechanism. This electrochemical behaviour is quite consistent with the discharge/charge voltage profiles of the 5 h electrode with small typical intercalation/de-intercalation plateaus (Figure 4d and Figure 5d).

### 3.3. Lithiation/De-Lithiation Behaviour

The nanoflakes of 5 h milled graphite with high BET specific surface area (350 m^2^ g^−1^) and fine particles exhibit lower specific capacity than CG, and 2 h milled graphite at a low to moderate current rates, but a superior rate performance of the 5 h electrode is realised when the current is changed to a higher density. Raman spectra of the CG and ball-milled graphite show that ball milling leads to producing structural defects in graphite. However, the significantly lower capacity and higher rate capability of the 5 h milled graphite cannot just be related to the increased disorder and defects in graphite structure because no noticeable differences are observed in the Raman spectrum between 2 h and 5 h milled graphite. Moreover, the enhanced crystallite boundaries in the particles of milled graphite (as shown by crystallite size decrease (Figure 1b) may cause their own effect on the lithiation behaviour of the powders as a structural defect. It will be discussed in the following analysis. The measured discharge capacities in the first cycle of the CG, 2 h, and 5 h electrodes are 396.4, 582.8, and 1478.7 mAh g^−1^, respectively. This significantly higher discharge capacity of the 5 h electrode in the first cycle (2.5 times of 2 h milled graphite) is compatible with the sharp rise of BET-specific surface area of the 5 h ball-milled graphite (Figure 1c). The high surface area can contribute to higher capacity because of the creation of more sites for Li^+^ intercalation. The large discharge capacity in the first cycle for ball-milled graphite electrodes with a higher surface area and finer graphite particles demonstrates that they are capable of higher Li^+^ storage. With respect to the diffusion formula described below, fine graphite powders produced by ball milling are beneficial for more intercalation of Li^+^ ions:

(2) $$\tau =L^{2}/D$$

where *τ* is the intercalation time, *D* is the diffusion coefficient, dependent upon the nature of the material, and *L* is the diffusion length, dependent upon the size of the material.

On the other hand, fine graphite particles with high surface area provide better access of the electrolyte to the bulk of the electrode, which leads to intense (electro)chemical reactions between them, followed by the formation of a larger SEI layer. It is confirmed by the big SEI peak in the CV curve (Figure 6c), and the high irreversible capacity of the 5 h milled graphite (Figure 4e). A significant number of Li ions are trapped inside the large SEI layer and cannot de-intercalate from the electrode [32,33,34]. Hence, finer active material particles with a high surface area can facilitate Li^+^ diffusion by shortening diffusion length; however, it develops a massive SEI layer, which prevents easy Li^+^ de-intercalation, and causes instability and a drop in capacity in the following cycles. Therefore, some strategies, such as modifying electrolytes and electrolyte additives [35,36], or developing effective techniques such as Li-doping [37], are needed to control the SEI layer impacts on the irreversible capacity of nanostructured graphite electrodes.

The electrochemical results show that when the surface area is doubled (2 h milled graphite), the rate capacity at 6 C, first cycle capacity, and irreversible capacity increase by ~42, 47, and 65% of CG, respectively. By increasing the surface area to ~35 times of CG (5 h milled graphite), the rate capacity at 6 C, first cycle capacity, and irreversible capacity increase to ~100, 273, and 700%, respectively. It demonstrates that the rise in the capacity is not proportional to the surface area increase. The increase in the irreversible capacity also reveals the impact of a large SEI layer formed on high-surface-area powders, which is a drawback of nanomaterials for LIBs and limits the LIB performance of the ball-milled graphite anodes [38].

Voltage profiles of 5 h milled graphite electrodes are almost sloppy and show a small plateau, while it is flat for 2 h milled graphite (Figure 4). Therefore, it can be inferred that Li^+^ ions do not intercalate easily in finer graphite particles (<180 nm), despite the fact that nanoscale particles facilitate the kinetics of Li^+^ ions diffusion and electron transport by shortening the diffusion pathways [39]; electrodes with finer graphite particles contain more graphite particles with ideal basal plane orientation (parallel with the direction of Li^+^ diffusion), which should be beneficial to Li^+^ ion insertion. The sloppy voltage profile may be explained by decreasing crystallite size from 32 nm for 2 h milled graphite to 11 nm for 5 h, which leads to an increasing volume fraction of crystallite boundaries. Crystallite boundaries can perform as the barriers for Li^+^ diffusion inside graphite particles due to different basal plane orientations in each crystallite that brings about unsmooth Li^+^ intercalation. More importantly, graphite particles with a high surface area can provide many potential surface places to absorb Li^+^ ions. If the Li^+^ ions absorbed on the surface of graphite particles exceed those that entered the particles, the main lithiation/de-lithiation mechanism cannot be intercalation/de-intercalation anymore, leading to a small or disappeared plateau.

Cyclic voltammograms of the 5 h milled graphite are unlike the CG and 2 h ones, as the intercalation peaks are minor in CV profiles of the 5 h sample, and de-intercalation peaks become lower. In addition, a few small peaks or distortions are observed throughout both lithiation and de-lithiation of the 5 h electrode. Voltage profiles of the 5 h electrode are also different from the CG and 2 h ones, showing nearly sloppy curves instead of flat profiles in low and moderate current rates and quite sloppy curves in high current rates. These can be a demonstration of a different lithiation/de-lithiation mechanism rather than typical Li^+^ ions intercalation/de-intercalation. The noticeable increase in first cycle capacity and rate capability of the 5 h electrode also shows activation of a different lithiation/de-lithiation mechanism in both slow and fast charge-discharge processes. The new mechanism should be dependent on a major structural change of the ball-milled graphite, i.e., BET-specific surface area, as it increases significantly from 9.75 to 349.57 m^2^ g^−1^ (~35 times) by extending ball milling to 5 h. This high surface area plays a major role in the new lithiation/de-lithiation mechanism.

**Figure 7:**
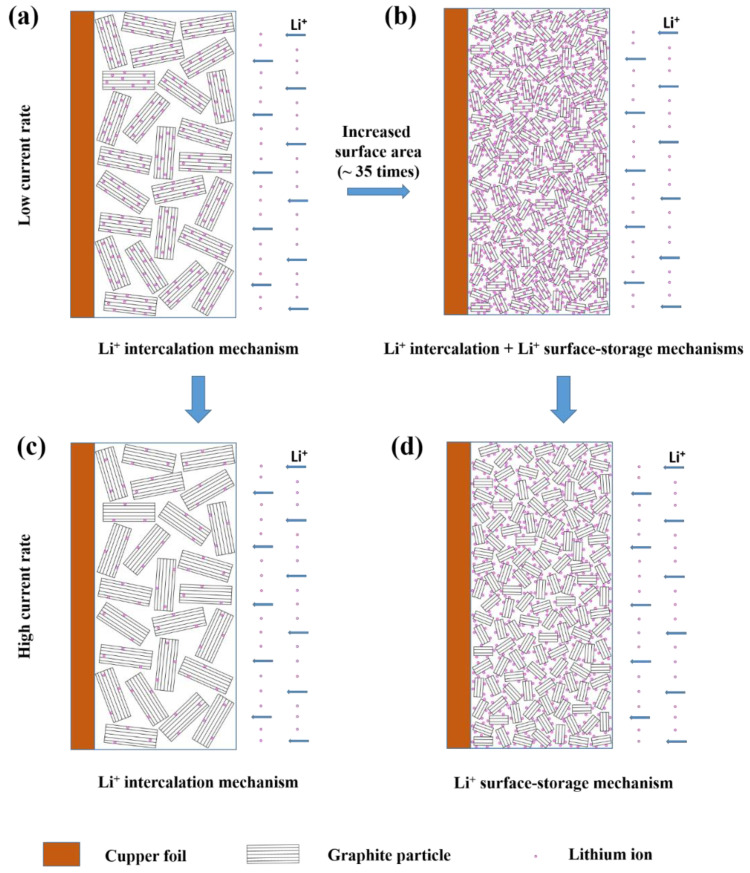
Schematic of the evolution in the lithiation mechanism of graphite electrode by increasing the surface area of graphite at low (**a**,**b**) and high current rates (**c**,**d**).

Figure 7 schematically represents how the lithiation/de-lithiation mechanism of graphite anodes can change at low and high current rates with increasing surface area of graphite. Figure 4a shows that increasing the surface area of graphite can lead to a high first discharge capacity by Li^+^ absorption on the surface of the particles (Figure 7b). Typical lithiation/de-lithiation mechanism (intercalation/de-intercalation) depends on the diffusion of Li^+^ ions into graphite, so it is a diffusion-controlled and time-dependent reaction. Since high charge-discharge rates do not provide the required time for Li^+^ ions to diffuse into the graphite particles, Li^+^ ions interact with the surface of graphite particles (Figure 7c,d). Therefore, this new mechanism can be Li^+^ surface-storage or Li^+^ surface absorption, which is dependent on the surface area of the active material. By increasing the current rate, two lithiation/de-lithiation mechanisms, i.e., Li^+^ intercalation/de-intercalation and Li^+^ surface-storage are balanced so that in high current rates of >1 C, the second mechanism is dominant in the 5 h ball-milled graphite electrode with the high surface area (Figure 7b,d).

The 2 h electrode has better rate capability than CG and shows a significant plateau in voltage profiles at high current rates. The CV of the 2 h electrode is also similar to CG, representing sharp intercalation/de-intercalation peaks. Therefore, the Li^+^ surface-storage mechanism is operating at high current rates; however, it cannot be the dominant mechanism in the 2 h electrode with graphite nanosheets of 140–800 nm and a surface area of 22 m^2^ g^−1^. SEM images of the 2 h ball-milled graphite show that the employed ball-milling conditions successfully led to the size reduction of large commercial graphite particles and exfoliated them into thin flakes of nanometre size. A continuous and smooth Li^+^ ions intercalation/de-intercalation takes place within these thin flakes, as the potential profile of the 2 h milled graphite electrode is flat and shows good reversibility at charge curves (Figure 4c). This is further supported by CV analysis as 2 h ball-milled electrode shows very strong and overlapping de-intercalation peaks in their anodic scan (Figure 6b). Therefore, electrodes of graphite nanosheets with a size distribution of 140–800 nm exhibit better electrochemical performance in terms of reversibility and rate capacity over CG electrodes (115 versus 90 mAh g^−1^ at 3 C).

## 4. Conclusions

This section is not mandatory but can be added to the manuscript if the discussion is unusually long or complex. Nanostructured graphite powders with high surface area produced by ball milling were tested to use as high-rate anode materials for lithium-ion batteries. The nanoflakes of 5 h milled graphite with BET-specific surface area of 350 m^2^ g^−1^ showed a specific capacity of 87 mAh g^−1^ at 6 C, 74.3 mAh g^−1^ at 10 C, and 55.7 mAh g^−1^ at 20 C. Electrochemical characterisations of graphite powders with 350 m^2^ g^−1^ surface area confirmed that a new mechanism is functioning for lithiation/de-lithiation of the electrodes rather than the Li^+^ intercalation/de-intercalation mechanism. Considering the extremely high surface area, this new mechanism can be Li^+^ surface-storage or Li^+^ surface-absorption. By increasing the current rate, a balance between Li^+^ intercalation and Li^+^ surface-storage mechanisms takes place so that Li^+^ surface-storage mechanism becomes the dominant mechanism when ball-milled graphite half-cells are charge-discharged fast. The prolonged ball milling of graphite leads to a drop in the specific capacity of the electrodes, which can be attributed to the high surface area of the ball-milled graphite leading to intense (electro)chemical reactions between electrode and electrolyte and the formation of a large SEI layer. Therefore, to control the SEI layer impacts on the irreversible capacity of nanostructured graphite electrodes, some strategies, such as modifying electrolytes and electrolyte additives or developing effective techniques, such as Li-doping, can be employed.

## Data Availability

Data sharing is not applicable to this article.

## Supplementary Materials

The following are available online at https://www.mdpi.com/article/10.3390/mi14010191/s1, Figure S1: TEM images of 5 h sample; Figure S2: Cycling stability of CG and ball-milled graphite for various durations at a 0.25 C rate (1 C = 372 mA g^−1^) up to 100 cycles (a), reversible and irreversible capacity as a function of BET specific surface area of the active material (b).
