# Supplementary material for: A Study on High-Rate Performance of Graphite Nanostructures Produced by Ball Milling as Anode for Lithium-Ion Batteries
Source: Micromachines (Basel). 2023 Jan 12;14(1):191. doi: 10.3390/mi14010191 (PMC9862907; doi:10.3390/mi14010191)
Supplement: Supplementary file 1 [file micromachines-14-00191-s001.zip › micromachines-2065564-supplementary.pdf]

Micromachines-2065564 Supplementary

Supplementary Materials: The following are available online at www.mdpi.com/xxx/s1, Figure S1: TEM images of 5h sample, Figure S2: Cycling stability of CG and ball-milled graphite for various durations at a 0.25C rate (1C = 372 mA g^{-1}) up to 100 cycles (a), reversible and irreversible capacity as a function of BET specific surface area of the active material (b).

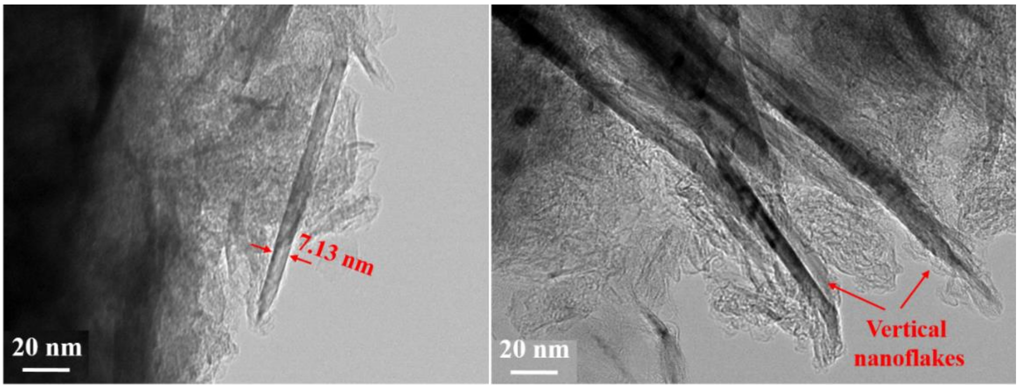

Figure S1. TEM images of 5h sample.

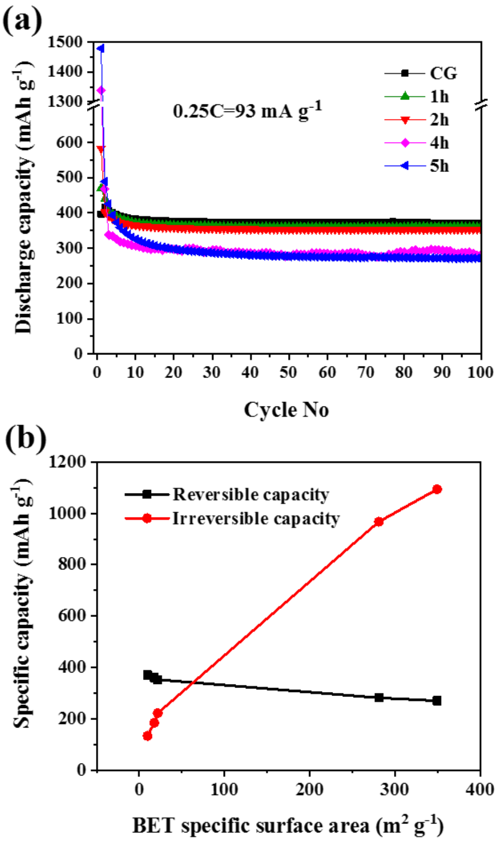

Figure S2. Cycling stability of CG and ball-milled graphite for various durations at a 0.25C rate (1C = 372 mA g^{-1}) up to 100 cycles (a), reversible and irreversible capacity as a function of BET specific surface area of the active material (b).
